# Cell envelope and stress-responsive pathways underlie an evolved oleaginous *Rhodotorula toruloides* strain multi-stress tolerance

**DOI:** 10.1186/s13068-024-02518-0

**Published:** 2024-05-28

**Authors:** Miguel Antunes, Marta N. Mota, Isabel Sá-Correia

**Affiliations:** 1 iBB – Institute for Bioengineering and Biosciences, Instituto Superior Técnico, Universidade de Lisboa, Av. Rovisco Pais, 1049-001 Lisbon, Portugal; 2 Department of Bioengineering, Instituto Superior Técnico, Universidade de Lisboa, Av. Rovisco Pais, 1049-001 Lisbon, Portugal; 3 Associate Laboratory i4HB – Institute for Health and Bioeconomy, Instituto Superior Técnico, Universidade de Lisboa, Av. Rovisco Pais, 1049-001 Lisbon, Portugal

**Keywords:** Oleaginous yeasts, Multi-stress tolerance, Stress responses, Functional genomics, Adaptive laboratory evolution, Cell wall

## Abstract

**Background:**

The red oleaginous yeast *Rhodotorula toruloides* is a promising cell factory to produce microbial oils and carotenoids from lignocellulosic hydrolysates (LCH). A multi-stress tolerant strain towards four major inhibitory compounds present in LCH and methanol, was derived in our laboratory from strain IST536 (PYCC 5615) through adaptive laboratory evolution (ALE) under methanol and high glycerol selective pressure.

**Results:**

Comparative genomic analysis suggested the reduction of the original strain ploidy from triploid to diploid, the occurrence of 21,489 mutations, and 242 genes displaying copy number variants in the evolved strain. Transcriptomic analysis identified 634 genes with altered transcript levels (465 up, 178 down) in the multi-stress tolerant strain. Genes associated with cell surface biogenesis, integrity, and remodelling and involved in stress-responsive pathways exhibit the most substantial alterations at the genome and transcriptome levels. Guided by the suggested stress responses, the multi-stress tolerance phenotype was extended to osmotic, salt, ethanol, oxidative, genotoxic, and medium-chain fatty acid-induced stresses.

**Conclusions:**

The comprehensive analysis of this evolved strain provided the opportunity to get mechanistic insights into the acquisition of multi-stress tolerance and a list of promising genes, pathways, and regulatory networks, as targets for synthetic biology approaches applied to promising cell factories, toward more robust and superior industrial strains. This study lays the foundations for understanding the mechanisms underlying tolerance to multiple stresses in *R. toruloides*, underscoring the potential of ALE for enhancing the robustness of industrial yeast strains.

**Supplementary Information:**

The online version contains supplementary material available at 10.1186/s13068-024-02518-0.

## Background

The basidiomycete yeast species *Rhodotorula toruloides* can accumulate lipids in the triacylglycerol form up to 70% of its dry cell weight, particularly in nutrient-limited environments [39, 41]. This “red” yeast also produces carotenoids and has a remarkable aptitude for utilizing a wide spectrum of carbon sources, including hexoses, pentoses, alcohols, organic acids, and long-chain fatty acids, being able to grow across a wide range of temperatures and pH [22, 39, 41, 64]. The *R. toruloides* strain IST536 (synonymous to *Rhodosporidium toruloides* CBS 6016 = IFO 8766 = MUCL 28631 = NBRC 8766 = NRRL Y-6987 = PYCC 5615) was selected in our laboratory for the valorization of sugar beet pulp (SBP) through lipid and carotenoid production by the complete utilization of all major carbon sources present in SBP hydrolysates, including D-galacturonic acid [37]. Being considered highly promising for industrial applications envisaging the transition to a sustainable circular bio-based economy, the increase of tolerance to relevant inhibitors present in lignocellulosic biomass hydrolysates represents an advantage for the exploitation of a wide-range of forest and agro-industrial residues as low-price sustainable feedstocks for industrial bioprocesses [10, 39, 41]. Those growth and metabolic inhibitors include weak acids, furan derivatives, and phenolic compounds. Despite the remarkable progress made recently, the available tools for *R. toruloides* genome engineering are still not highly efficient [64]. An Adaptive Laboratory Evolution (ALE) strategy was successfully used in our laboratory to enhance *R. toruloides* IST536 tolerance under selective pressure imposed by increasing inhibitory methanol concentrations added to a minimal medium with 5% (v/v) of glycerol as the sole carbon source [15]. This evolved strain, IST536 MM15, was found to be more tolerant not only to methanol but also to acetic and formic acids, furfural and 5-hydroxymethylfurfural (HMF), the four major inhibitors expected to be present in lignocellulosic biomass hydrolysates [15].

Despite the potential of *R. toruloides* as a microbial cell factory, its comprehensive genomic and physiological characterization remains limited. The availability of strains that can withstand various bioprocess-related stress conditions, is required [11, 31, 57] and the highly interesting traits of the evolved strain *R. toruloides* IST536 MM15 provides the opportunity to explore this strain, not only for potential biotechnological applications, but also to get clues into the multi-stress tolerance phenomenon. This study was designed to elucidate the underlying mechanisms through comparative functional genomic analyses of *R. toruloides* IST536 and its evolved counterpart, IST536 MM15. These strains were preliminary characterized in our laboratory and results indicate that the evolved multi-stress tolerant strain has a cell wall less susceptible to zymolyase activity and a decreased permeability, based on the intracellular accumulation of propidium iodide fluorescent probe [15]. The registered alteration of the cell envelope properties guarantees a robust adaptive response to stress. In the particular case of the weak acids, acetic and formic acids, this alteration may also limit the futile cycle associated with the re-entry of the acid form after the active expulsion of the counter-ion from the cell interior [42, 50]. However, the identification of key genes at a genome-wide scale and global mechanisms responsible for the multiple stress tolerance features of IST536 MM15 is still lacking. To achieve a comprehensive understanding of the global alterations occurring at the genome and transcriptome levels in the evolved strain compared with the original strain, their genome sequences and transcription levels were compared under non-stressing conditions. This genome-wide analysis provided useful insights into the mechanisms underlying multi-stress tolerance, underscoring the potential of ALE in enhancing the robustness of industrial yeast strains.

## Results

### Genome analysis of the evolved multi-stress tolerant strain compared to the original strain

**Fig. 1:**
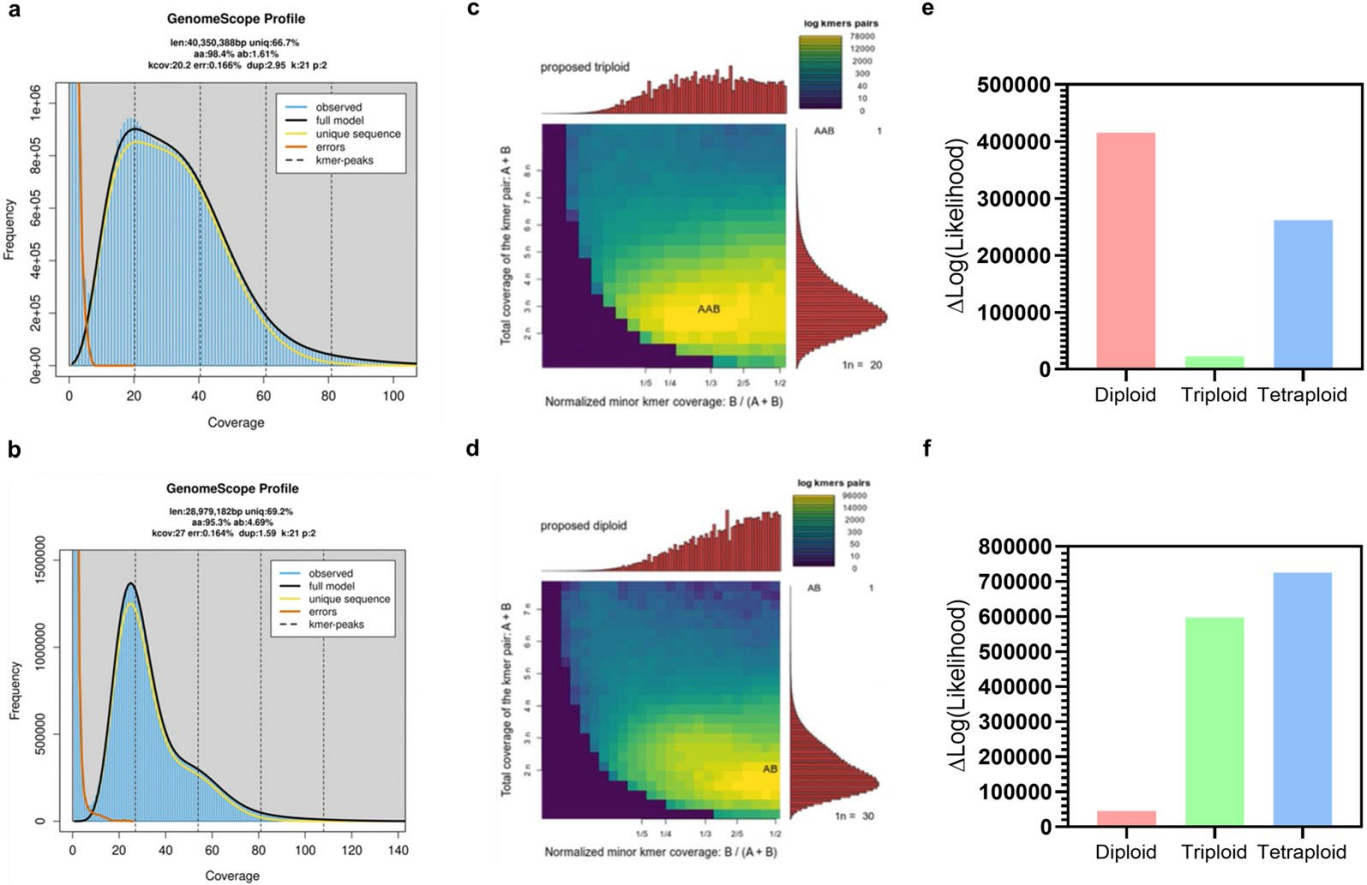
Estimation of *R. toruloides* IST536 and IST536 MM15 genome sizes and ploidy. k-mer distribution curves (k=21) generated with GenomeScope2.0 using Illumina reads for IST536 (**a**) and IST536 MM15 (**b**). Smudgeplots depicting ploidy predictions for IST536 (**c**) and IST536 MM15 (**d**). The estimated ploidies are displayed in the upper left corner of each plot. Different colour intensities indicate the approximate amount of k-mers per bin, which varies from purple (lower amount) to yellow (higher amount). Barplots depicting ploidy estimations using nQuire for the IST536 (**e**) and IST536 MM15 (**f**) strains based on $\Delta$log-likelihood values of the three fixed models (diploid, triploid, and tetraploid)

Genome profiling with Illumina data using GenomeScope2.0 estimated the strain IST536 to be a triploid and the strain IST536 MM15 to be a diploid (Fig. 1c, d). These estimations were concordant with the nQuire estimates, which resulted in $\Delta$log-likelihood values lower in the triploid model for IST536 and in the diploid model for IST536 MM15 (Fig. 1e, f). In nQuire, if a model has a lower $\Delta$log-likelihood value, it means it is a better fit for the frequency distribution of variant sites from aligned reads. Thus, the assumed ploidy of the sample is the ploidy model with the lowest $\Delta$log-likelihood value. The results from nQuire for the first 100 scaffolds of IST536 genome assembly indicate that overall, it has a triploid genome, showing significant levels of aneuploidy (Additional File 1; Supplementary Fig. 3A), whereas for IST536 MM15 most of the genomes appears to be diploid, while displaying a few aneuploidies (Additional File 1; Supplementary Fig. 3B). In addition, both strains displayed high estimated heterozygosity; 1.61% for IST536 and 4.69% for IST536 MM15 (Fig. 1a and b). Given the high levels of heterozygosity, commonly found in hybrid species, *de novo* assembly was performed using SPAdes in combination with Redundans, which discards uncollapsed contigs through a reducing step, resulting in the construction of contigous chimeric assemblies. The final genome assembly sizes (Additional File 1; Supplementary Table 1) were identical to previously described *R. toruloides* strains. GenomeScope2.0 estimated a genome size of 40 Mbp for IST536 (Fig. 1a), consistent with the size originally derived through the initial assembly via SPAdes (41 Mbp), which, after reduction by removal of redundant contigs, was determined to be 23 Mbp (Additional File 1; Supplementary Table 1). Gene prediction guided by RNA-seq data resulted in the annotation of 8480 genes and 8291 transcripts, with an average of 6.57 exons per gene. The region of the genome assembly containing the IST536 mitochondrial genome was identified based on scaffold-wide depth of coverage assessment. Scaffold70 exhibited significant high coverage, subsequently confirmed to correspond to the mitochondrial genome through blastp analysis. An annotated map for the mitochondrial genome is illustrated in  Additional File 1; Supplementary Fig. 1. The mitochondrial draft genome has a length of 114 kb and a GC content of 45%, which agrees with the values reported for the mitochondrial genomes of *R. toruloides* NP11 and IFO 0880 [65]. The lower GC content of the mitochondrial genome compared to the nuclear genome (62%) is noted as a common phenomenon in fungi [65].

A total of 21,489 variants were found, with 14,571 representing single nucleotide polymorphisms (SNPs). The categorical distribution and genomic regions affected by these variants are summarized in Additional File 1; Supplementary Fig. 2. For further analyses, missense variants (28%) and non-sense variants (0.3%) were selected (Additional file 2). Notably, these mutations were selected due to their considered high or moderate impact. High-impact mutations are anticipated to substantially disrupt protein function, while moderate-impact mutations are expected to alter protein activity in a manner that does not necessarily lead to disruption. Missense variants were identified in 1,115 genes, while non-sense variations were found in 25 genes. Among the genes harbouring missense variants, 30% encode hypothetical proteins.

**Fig. 2:**
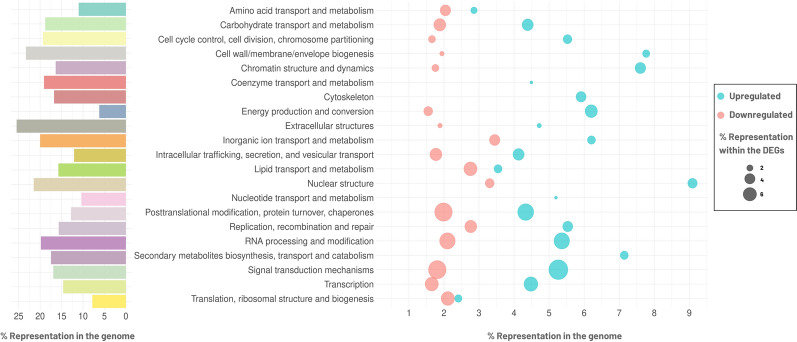
Functional Enrichment Analysis of missense or non-sense gene variants and differentially expressed genes (DEGs) in the evolved strain versus the parental strain. The barplot illustrates the distribution of genes across the functional categories relative to the total number of genes within *R. toruloides* IST536 genome. The dot plot illustrates the distribution of the genes with increased transcription levels (referred as upregulated; in blue) and with decreased transcription levels (referred as downregulated; in pink) genes during the exponential growth phase across the functional categories relative to the total number of genes within the *R. toruloides* genome. The percentage of DEGs assigned to each category relative to the total number of DEGs in the data set is represented by the dot sizes. Functional categories are based on EuKaryotic Orthologous Groups (KOG) annotations

To provide a comprehensive understanding of the functional implications derived from the identified mutations, a classification of the genes harbouring non-synonymous mutations was undertaken based on KOG categories. This analysis considered the percentage of genes assigned to each functional category relative to the overall percentage of genes in the genome belonging to that specific functional category (Fig. 2). The distribution of genes exhibits a notable enrichment in categories associated with extracellular structures, cell wall/membrane/envelope biogenesis, nuclear structures, inorganic ion transport and metabolism, and RNA processing and modification as the top five.

Considering the genes identified as encoding transporter proteins, approximately 20% are predicted to encode multidrug resistance (MDR) transporters. These genes include IST_001210 (*QDR2/QDR1/AQR1*), IST_002315 (*QDR3*), IST_006965 (*TPO1*), and IST_007245 (*TPO2/TPO1/TPO3*), encoding putative transporters from the major facilitator superfamily (MFS) from the drug:(H^+^) antiporter DHA family, and IST_007245 (*SNQ2/PDR18/PDR12*), encoding a putative ATP-binding cassette (ABC) transporter. The activity of these putative plasma membrane efflux pumps involved in MDR are among the proposed mechanisms used by yeast to overcome deleterious effects resultant from chemical stresses; presumably through active export of a significantly wide spectrum of structurally unrelated hydrophobic compounds [52, 53]. Due to their high homology and apparent redundancy [52, 53], it was not possible to associate a single *S. cerevisiae* orthologous gene to three *R. toruloides* ORFs.

A comprehensive search to identify protein kinases and transcription factors harboring missense mutations and with an *S. cerevisiae* orthologue was conducted and summarized in Tables 1 and 2, respectively. This focus is justified by the crucial roles these proteins play in cellular function and regulation since transcription factors are integral to the regulation of gene expression and kinases are critical regulators of cell signalling pathways, including the regulation of transcription factors activity [63]. Genomic alterations in these proteins may alter the expression of target genes and protein phosphorylation patterns. Among the genes encoding putative kinases, two members from the cell wall integrity (CWI) pathway in *S. cerevisiae* were identified: Pkc1 and Bck1. Pck1 plays a crucial role in initiating the mitogen-activated protein kinase (MAPK) cascade, comprised of Bck1, Mkk1/2 and Slt2, in response to cell wall stress [27, 50]. This cascade is pivotal for cell wall remodeling. In addition, kinases associated to the coordination of the DNA damage response and cell cycle checkpoint pathways in *S. cerevisiae*, such as Mec1, Tel1, Gnc2, and Yox1 [8, 46] were also identified. Regarding transcription factors, missense mutations were identified in putative regulators of stress responses, including Hsf1, a trimeric heat shock transcription factor activated in response to a variety of environmental stress factors, Rox1, a heme-dependent transcription factor with roles in the response to oxidative and hyperosmotic stress, Cup2, involved in the regulation of copper homeostasis genes in response to DNA damage and oxidative stress, and Hcm1, implicated in the activation of genes related to respiration and response to oxidative stress, and whose regulation is influenced by the cell wall integrity checkpoint (as described in https://www.yeastgenome.org/).

**Table 1:** Genes predicted to encode protein kinases with identified missense variants in the evolved strain IST536 MM15 compared to the parental strain IST536

ORF	*S.cerevisiae* Orthologue	Variants	Description
IST_000174	*ALK1*	1	Required for proper spindle positioning and nuclear segregation following mitotic arrest and proper organization of cell polarity factors in mitosis
IST_002379	*AVO1*	2	Component of a membrane-bound complex containing the Tor2p kinase
IST_006033	*BAS1*	2	Involved in regulating basal and induced expression of genes of the purine and histidine biosynthesis pathways; also involved in regulation of meiotic recombination at specific genes
IST_000908	*BCK1*	1	MAPKKK acting in the protein kinase C signalling pathway; the kinase C signalling pathway controls cell integrity
IST_002150	*CCL1*	1	Cyclin partner of the cyclin-dependent kinase (CDK) Kin28p; regulates the activity of Kin28p
IST_005946	*CDC15*	1	Hippo-like kinase of the Mitotic Exit Network; promotes exit by activating the Dbf2p kinase
IST_004845	*FBP26*	2	Fructose-2,6-bisphosphatase, required for glucose metabolism
IST_003456	*GCN2*	2	Phosphorylates the alpha-subunit of translation initiation factor eIF2 (Sui2p) in response to starvation
IST_000210	*KIN2*	4	Regulates polarized exocytosis and the Ire1p-mediated UPR; regulates *HAC1* mRNA translocation, splicing and translation with *KIN1* during ER stress; may regulate septin and cell wall organization
IST_007802	*LCB5*	1	Minor sphingoid long-chain base kinase; possibly involved in synthesis of long-chain base phosphates, which function as signaling molecules
IST_001552	*MDG1*	4	Involved in G-protein mediated pheromone signalling pathway
IST_000141	*MEC1*	2	Signal transducer required for cell cycle arrest and transcriptional responses to damaged or unreplicated DNA
IST_007422	*PIK1*	3	Phosphatidylinositol 4-kinase; catalyses the first step in the biosynthesis of phosphatidylinositol-4,5-biphosphate; required for autophagosome formation during autophagy and for lipophagy in both stationary phase cells and during nitrogen starvation
IST_008422	*PKC1*	1	Essential for cell wall remodelling during growth
IST_003463	*PYK2*	1	Pyruvate kinase; appears to be modulated by phosphorylation; transcription repressed by glucose
IST_002496	*RBK1*	1	Ribokinase, required for recycling ribose during nucleotide metabolism
IST_000527	*SAK1*	1	Upstream serine/threonine kinase for the *SNF1* complex
IST_002932	*SEC59*	1	Dolichol kinase; required for viability and for normal rates of lipid intermediate synthesis and protein N-glycosylation
IST_003305	*SGV1*	1	Part of the BUR kinase complex which functions in transcriptional regulation; regulated by Cak1p
IST_004129	*SNF4*	3	Activating gamma subunit of the AMP-activated Snf1p kinase complex
IST_006498	*TEL1*	2	Protein kinase primarily involved in telomere length regulation; contributes to cell cycle checkpoint control in response to DNA damage

The orthologue genes in *S. cerevisiae* are displayed along with their functional description retrieved from the *Saccharomyces* Genome Database (SGD)—https://www.yeastgenome.org/

**Table 2:** Genes predicted to encode transcription factors with identified missense variants in the evolved strain IST536 MM15 compared to the parental strain IST536

ORF	*S.cerevisiae* Orthologue	Variants	Description
IST_005150	*ARG81*	1	Involved in the regulation of arginine-responsive genes
IST_006521	*COM2*	3	May bind the *IME1* promoter under all growth conditions to negatively regulate its transcription in the absence of a positive regulator that binds more effectively; repressor activity may depend on phosphorylation by PKA
IST_002009	*CUP2*	3	Activates transcription of the metallothionein genes *CUP1-1* and *CUP1-2* in response to elevated copper concentrations; required for regulation of copper genes in response to DNA-damaging reagents
IST_000080	*DAL80*	1	Negative regulator of genes in multiple nitrogen degradation pathways
IST_002777	*HCM1*	1	Drives S-phase activation of genes involved in chromosome segregation, spindle dynamics, budding; also activates genes involved in respiration, use of alternative energy sources, NAD synthesis, oxidative stress resistance; regulated by cell wall integrity checkpoint
IST_002263	*HSF1*	4	Trimeric heat shock transcription factor; activates multiple genes in response to highly diverse stresses and transient intracellular acidification; posttranslationally regulated
IST_000209	*ROX1*	1	Heme-dependent repressor of hypoxic genes; repressor function regulated through decreased promoter occupancy in response to oxidative stress; involved in the hyperosmotic stress resistance
IST_003369	*RRN3*	1	Protein required for transcription of rDNA by RNA polymerase I
IST_000205	*SFP1*	1	Regulates transcription of ribosomal protein and biogenesis genes; regulates response to nutrients and stress, G2/M transitions during mitotic cell cycle and DNA-damage response, and modulates cell size
IST_003236	*SPT8*	2	Subunit of the SAGA transcriptional regulatory complex; not present in SAGA-like complex SLIK/SALSA; required for SAGA-mediated inhibition at some promoters
IST_003340	*STB4*	3	Putative transcription factor; computational analysis suggests a role in regulation of expression of genes encoding transporters
IST_007166	*TEC1*	2	Transcription factor targeting filamentation genes and Ty1 expression; Ste12p activation of most filamentation gene promoters depends on Tec1p and Tec1p transcriptional activity is dependent on its association with Ste12p
IST_006138	*UGA3*	1	Transcriptional activator for GABA-dependent induction of GABA genes
IST_002548	*URE2*	3	Nitrogen catabolite repression transcriptional regulator; inhibits *GLN3* transcription in good nitrogen source
IST_003829	*YAP6*	3	Physically interacts with the Tup1-Cyc8 complex and recruits Tup1p to its targets; overexpression increases sodium and lithium tolerance
IST_002119	*YOX1*	1	Homeobox transcriptional repressor; binds to Mcm1p and to early cell cycle boxes (ECBs) in the promoters of cell cycle-regulated genes expressed in M/G1 phase; phosphorylated by Cdc28p
IST_001178	*YRR1*	1	Activates genes involved in multidrug resistance

The orthologue genes in *S. cerevisiae* are displayed along with their functional description retrieved from the *Saccharomyces* Genome Database (SGD)—https://www.yeastgenome.org/

**Fig. 3:**
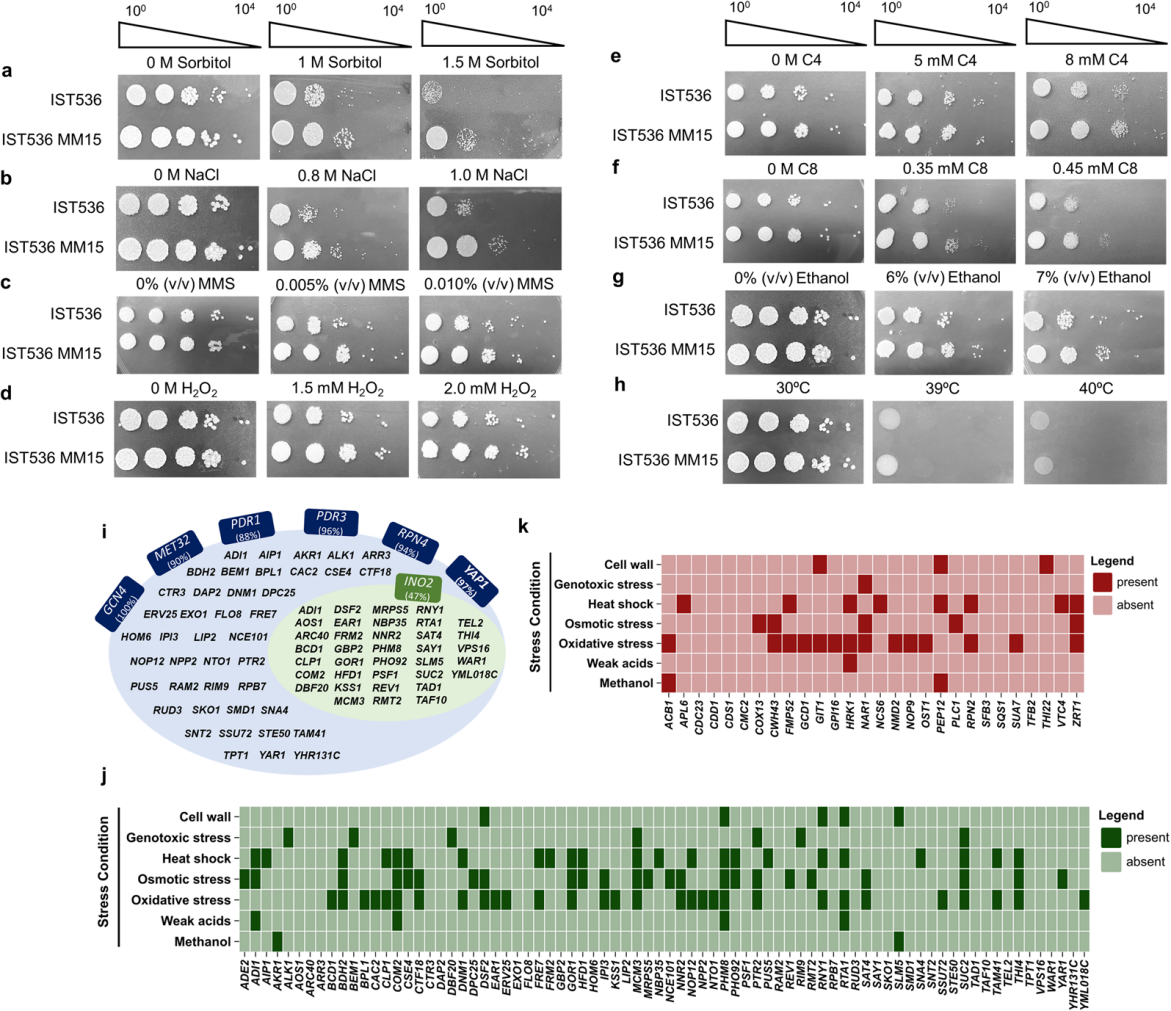
Stress tolerance and regulatory associations between transcription factors and upregulated target genes in the evolved versus the original strain. Susceptibility assays of *R. toruloides* strains IST536 and IST536 MM15 to diverse stress conditions. Growth of the evolved and parental strains in YPD media supplemented, or not, with 1.0 and 1.5 M sorbitol (**a**), 0.8 and 1.0 M sodium chloride (NaCl; **b**), 0.005 and 0.010% (v/v) methyl methanesulfonate (MMS; **c**), 1.5 and 2.0 mM hydrogen peroxide (H_2_O_2_; **d**), 5.0 and 8.0 mM butyric acid (C4; **e**), 0.35 and 0.45 mM octanoic acid (C8; **f**), and 6.0 and 7.0% (v/v) ethanol (**g**). Susceptibility phenotypes were registered after 72 h of incubation at 30^∘^C. For the thermotolerance assay, YPD media plates were incubated at 30^∘^C (control), 39^∘^C and 40^∘^C (**h**). The displayed results are representative of equivalent results obtained from at least two independent experiments. **i** Transcription factors predicted to regulate the genes exhibiting increased transcriptional levels in *R. toruloides* IST536 MM15. Only the genes with an *S. cerevisiae* orthologue and not displaying a copy number gain were considered for the analysis. The identification of these transcription factors was based on the registered transcriptional regulatory associations for *S. cerevisiae* in Yeastract database. The percentage of potentially regulated genes within the used dataset was determined through the *RankByTF* tool from Yeastract. (**j**) Distribution of the genes displaying increased transcriptional levels and absence of copy number variations into stress conditions under which their transcriptional levels are documented to be influenced. (**k**) Distribution as in panel **j** of the genes displaying increased transcriptional levels and an increase in copy number

The determination of copy number variants is also instrumental to interpret alterations registered at the transcriptional levels, as discussed in other sections. The copy number variants between the evolved strain and the parental strain were assessed based on a per gene depth of coverage approach. Overall, 182 genes were considered to have an increase in copy number, whereas 60 genes were considered to have a reduction (Additional file 3). Genes displaying an apparent alteration in copy number exhibit diverse functions, such as chromatin remodelling, intracellular vesicular trafficking, cellular transport, and regulation of the unfolded protein response. Notably, the gene orthologous to *S. cerevisiae* *ENA2* exhibits a gain in copy number as well as a missense variant. Copy number variation of *ENA* genes is documented to correlate to NaCl tolerance in *S. cerevisiae* [55]. Guided by this information, hyperosmotic and saline stress tolerance was evaluated by exposing cells to high concentrations of the non-metabolizable alcohol, sorbitol (Fig. 3a), and to elevated concentrations of NaCl (Fig. 3b). The evolved strain displayed indeed higher tolerance to both sorbitol- and NaCl-induced stresses compared to the original strain. Another example is the gene orthologous to *S. cerevisiae* *PCL1*. In *S. cerevisiae*, *PCL1*, localized within a sub-telomeric region and thus susceptible to copy number variation [7], has been implicated in the modulation of nutrient sensing, filamentous growth, and PKA-mediated stress response.

### Transcriptional profile of the evolved multi-stress tolerant *R. toruloides* strain compared to the original strain

The identified Differentially Expressed Genes (DEGs) reflect cell alterations at the transcriptional level that occur in the evolved strain. A total of 634 DEGs were identified among the 8480 predicted genes, using as cutoff values a fold-change $\ge$ 2.0 and an FDR $\le$ 0.05 (Additional File 4). Twenty four percent of these genes encode “hypothetical proteins”. Among the identified DEGs, 456 genes displayed significantly increased mRNA levels, while 178 genes exhibited lower mRNA levels. Among the genes with increased transcript levels, 6.4% exhibited non-synonymous variants, while 22% demonstrated a gain in copy number. Conversely, 17.4% of the genes with decreased transcript levels were associated with non-synonymous variants, and 6.7% exhibited a loss in copy number. To retrieve the biological functions of each ORF, the genome annotations for *R. toruloides* strains NP11, IFO 0559, and IFO 0880 retrieved from the Joint Genome Institute’s (JGI) Mycocosm database (https://mycocosm.jgi.doe.gov/mycocosm/home) [40], were used. To enhance the inference of potential gene functions, the orthologous genes in *R. toruloides* NP11, IFO 0559, and IFO 0880, and in *S. cerevisiae* for each *R. toruloides* IST536 ORF were inferred using Orthofinder [13]. Notably, 70% of the identified DEGs lack associated orthologous genes in *S. cerevisiae*, which is consistent with the phylogenetic distance between these species.

A comprehensive functional enrichment analysis was performed using the Eukaryotic Orthologous Groups (KOG) annotation data sets for *R. toruloides* NP11, IFO 0559 and IFO 0880 to assess the distribution of DEGs assigned to each functional class in relation to the total number of genes within the *R. toruloides* IST536 genome encompassing each respective functional class (Fig. 2). The most abundant functional categories among the genes associated with increased transcription levels in the evolved multi-stress tolerant strain are “Cell wall/envelope/membrane biogenesis”, followed by “Chromatin structure and dynamics”, and “Secondary metabolites biosynthesis, transport and catabolism” (Fig. 2). Conversely, the most abundant functional categories among the genes associated with decreased transcription levels in the evolved strain are “Inorganic ion transport and metabolism”, “Replication, recombination and repair”, and “Lipid transport and metabolism” (Fig. 2). Remarkably, the most abundant functional categories from the genes displaying increased or decreased transcript levels, were categories also observed as relevant for gene variants (Fig. 2). The functional categories “Cytoskeleton”, “Secondary metabolites biosynthesis, transport and catabolism”, “Nucleotide transport and metabolism”, and “Coenzyme transport and metabolism” do not include any genes displaying decreased transcription levels. The above referred untargeted functional analysis was complemented with an analysis of the relevant transcriptional alterations based on the functional annotations of *R. toruloides* IFO 0559, IFO 0880 and NP11, and the inferred orthologues in *S. cerevisiae*. The DEGs classified as encoding “hypothetical proteins” were not included in this analysis.

The evolved strain exhibits a notable increase in transcription levels across various genes associated with iron and copper metabolism. Specifically, three genes potentially encoding iron reductases, a complex transport system for iron, and genes involved in the cytosolic assembly of Fe–S clusters (*NAR1* and *NBP35*), along with their subsequent incorporation into proteins (*IBA57*), demonstrated a significant increased mRNA levels. In addition, the transcription levels from three genes possibly responsible for copper transport were found to be elevated. In yeast, copper and iron metabolism are interconnected since copper is a cofactor for enzymes involved in iron metabolism [48]. Regarding transporter encoding genes, there is an increase in the transcriptional levels of transporters related to sugar transport (glucose, galactose, and fructose), a glycerol:H^+^ symporter (with similarity to *S. cerevisiae* Stl1), a potassium transporter (with similarity to *Debaryomyces occidentalis* Hak1), and transporters with similarity to *S. cerevisiae* Ptr2 (peptide:H^+^ symporter), Mch5 (riboflavin transporter), and Fui1 (uridine permease). Transporter proteins with decreased transcriptional levels include two maltose transporters, two amino acid transporters, a potassium uniporter (Trk1/2), vacuolar Mn^2+^,Ca^2+^:H^+^ and Zn^2+^,Cd^2+^:H^+^ antiporters, an allantoate transporter (Dal5), a quinate:H^+^ symporter, a pantothenate:H^+^ symporter. and a phospholipid flipping ATPase (Neo1). Increased transcription levels were also found from genes that collectively influence lipid metabolism, transport and signalling. For instance, genes involved in phospholipid synthesis include a CDP-diacylglycerol synthase (*CDS1*, displaying a copy number gain), which takes center stage in phospholipid synthesis; a phosphatidic acid phosphatase type 2, which converts phosphatidic acid to diacylglycerol—a precursor required for the synthesis of both phospholipids and triacylglycerol; and a mitochondrial CDP-diacylglycerol synthase (*TAM41*), which plays a pivotal role in the synthesis of the phospholipid cardiolipin required for the maintenance of mitochondrial membrane integrity. Genes involved in phospholipid transport included a phosphotidylinositol transfer protein and a phosphatidylethanolamine-binding protein, which may be involved in the transport of phospholipids within the cell. Furthermore, genes encoding enzymes involved in the $\beta$-oxidation of fatty acids and hydrolysis of acylglycerols, and in the cleavage of carotenoids were also found to have heightened transcript levels, suggesting metabolic alterations influencing lipid mobilization and cellular energy balance. Several genes encoding proteins involved in DNA repair (e.g., *DIN7, CTF18, REV1*) were also found to have increased transcriptional levels in the evolved strain. Differential expression analysis also revealed an increase in the transcription levels from genes implicated in cell wall structure and integrity. Considering the importance of these genes in this study context, the identified alterations at different levels are separately detailed.

#### Involvement of cell surface synthesis and remodeling genes in the multi-stress tolerance phenotype of the evolved strain

Enhanced transcriptional activity and genomic alterations were consistently observed in cell wall-associated genes in the evolved strain, in comparison to the parental strain. These genes are involved in various functions, including protein glycosylation, $\beta$-glucan biosynthesis and assembly, and chitin metabolism. Notably, genes linked to glycosylphosphatidylinositol (GPI) anchors, such as a probable GPI lipid remodelase (*CWH43*), and a subunit of the GPI transaminase complex (*GPI16*), exhibit increased transcription levels, possibly due to amplified copy numbers (Additional File 4). Furthermore, genes encoding GPI biosynthesis proteins (*GPI1*, *GPI13*, and *SMP3*), along with those involved in attaching GPI anchors to proteins (*GAB1*, *GPI17*), were found to possess several missense variants. The importance of GPI anchors in mediating the attachment of mannoproteins to the cell wall outer layer is well-documented [16, 50]. Several plasma membrane GPI-anchored proteins have been shown to display transglycosylase activities contributing to cell wall remodelling [23, 50].

In addition, genes related to $\beta$-glucan biosynthetic pathways, such as *KRE5* (essential for $\beta$-1,6 glucan biosynthesis), *CWH41*, and *EXG1* (encoding a processing $\alpha$-glucosidase I and a major exo-1,3-$\beta$-glucanase, respectively), were observed to contain numerous missense variations. These genes are crucial in $\beta$-glucan assembly within the cell wall. Alterations were also detected in genes associated with glycosylation, including *PSA1* (a GDP-mannose pyrophosphorylase with a missense variation), involved in cell wall biosynthesis, and mannosyltransferases (*MNN2/5*, *ALG2*, *ALG3*, and *ALG12*), with changes noted at both transcriptional and genomic levels. Protein mannosylation has been described as playing a notable role in cell wall integrity and endoplasmic reticulum (ER) homeostasis in yeast [6].

Oleaginous yeast species, such as *R. toruloides*, differ from non-oleaginous yeasts like *S. cerevisiae* in having higher chitin and mannan content in their cell walls, contributing to increased rigidity and resistance to lysis [25]. In the examined evolved strain, a significant increase in the abundance of transcripts and genomic variants of genes encoding chitin-modifying enzymes (e.g. chitin synthase *CHS1*) was noted, underscoring their significance in the modulation of cell wall chitin content in the evolved strain. Furthermore, genomic variations in *PKC1* and *BCK1* were identified in the evolved strain. In *S. cerevisiae*, the Pkc1-MAPK cascade, a primary Rho1 effector pathway, is crucial for cell wall synthesis regulation, besides its roles in heat stress response, hypotonic shock adaptation, and actin cytoskeleton organization [51].

### Prediction of altered putative regulatory networks in the evolved multi-stress tolerant strain

To get insights into the mechanisms behind putative transcriptional networks altered in IST536 MM15 compared to IST536, two different approaches were applied considering the genes identified as concomitantly exhibiting increased transcript levels and absence of a copy number increase. The first approach was based on the analysis of motifs present in the promoter sequences of these genes. The identification of transcription factors whose DNA-binding motifs are enriched in the promoter sequences of genes exhibiting increased transcriptional levels and without an increase in copy number, was conducted. Promoter sequences (-1000 bp) of these genes were extracted and utilized for motif prediction. The transcription factors corresponding to the candidate transcription factor binding sequences (TFBS) were identified and the percentage of genes potentially regulated by these TFs, based on the documented transcriptional associations for *S. cerevisiae* in YEASTRACT+ [58], were determined using the *RankByTF* tool. Since the identification of putative transcription factors relied on the DNA-binding motifs present in the JASPAR fungi database, which in this case resulted solely in matches to *S. cerevisiae* transcription factors, it is likely that some of these transcriptional regulators may lack ortologous genes in *R. toruloides*. The identified putative transcriptional regulators are mainly associated with amino acid biosynthesis and catabolism (*LEU3*, *CHA4*), cytokinesis (*ACE2*) and DNA damage-induced responses and telomere length maintenance (*RFX1*, *RAP1*, *MIG3*) (Table 3). The latter suggested response is in agreement with the increased tolerance in the evolved strain compared to the original strain to the alkylating agent MMS (Fig. 3c).

**Table 3:** Transcription factors with enriched DNA-binding motifs in the promoters of genes displaying increased transcriptional levels in the evolved strain

*S. cerevisiae* Transcription factor	*R. toruloides* IST536 TF Orthologue	Description	Potentially regulated genes (%)
Ace2	IST_006265	Transcription factor required for septum destruction after cytokinesis; part of the RAM network that regulates polarity and morphogenesis	12.0
Cha4	IST_005286	Mediates serine/threonine activation of the catabolic L-serine (L-threonine) deaminase (*CHA*1)	3.0
Leu3	–	Repressor and activator; regulates genes involved in branched chain amino acid biosynthesis and ammonia assimilation; acts as a repressor in leucine-replete conditions and as an activator in the presence of alpha-isopropylmalate, an intermediate in leucine biosynthesis that accumulates during leucine starvation	12.0
Mig3	–	Partially nonfunctional in S288C strains but has a major role in catabolite repression and ethanol response in some other strains; involved in response to toxic agents; phosphorylation by Snf1p or the Mec1p pathway inactivates Mig3p, allowing induction of damage response genes	21.0
Rap1	–	Essential DNA-binding transcription regulator that binds many loci; involved in transcription activation, repression, chromatin silencing, telomere length maintenance	42.0
Rfx1	–	Major transcriptional repressor of DNA-damage-regulated genes; involved in DNA damage and replication checkpoint pathway	11.0
Swi5	IST_006265	Transcription factor that recruits Mediator and Swi/Snf complexes; activates transcription of genes expressed at the M/G1 phase boundary and in G1 phase; required for expression of the HO gene controlling mating type switching	5.0

The identified *S. cerevisiae* transcription factors and the respective orthologues in *R. toruloides* IST536 and NP11 are displayed. Functional descriptions were obtained from the *Saccharomyces* Genome Database (SGD)—https://www.yeastgenome.org/. The percentage of potentially regulated genes was determined using *RankByTF* tool from Yeastract

The second approach consisted of a search for transcription factors based on the documented transcriptional regulatory associations for *S. cerevisiae* in the Yeastract database using, among the aforementioned genes with increased transcript levels and without identified increase in copy number, only those with orthologous genes in *S. cerevisiae*. The tool *RankByTF* was again employed to identify putative transcription factor networks, considering the database transcriptional regulatory associations under both unstressed and stressed conditions (Fig. 3i). This approach was taken because, although the transcriptional alterations in the evolved strain were obtained under unstressed conditions, the genomic changes are a result of an adaptive laboratory evolution (ALE) process under stress conditions. The significant TFs identified include Gcn4, an activator of amino acid biosynthetic genes; Met32, a regulator of methionine biosynthetic and sulfur metabolism genes; Ino2, a regulator of phospholipid biosynthetic genes; Yap1, central to oxidative stress response; Pdr1/3, modulators of the pleiotropic drug resistance network; and Rpn4, which influences proteasome-mediated protein catabolism and DNA repair (Fig. 3i). The genes used for this analysis were distributed across the stress conditions under which their transcriptional levels are documented to be influenced (Fig. 3j). The same process was applied to the genes both exhibiting increased transcription levels and copy numbers (Fig. 3k). Considering the genes without copy number alterations, a substantial fraction is associated to oxidative, osmotic and thermal stresses response, whereas genes with increased copy numbers are mainly associated with oxidative stress response. Susceptibility assays demonstrated that the evolved strain also exhibits increased tolerance, compared to the parental strain, to H_2_O_2_-induced oxidative stress (Fig. 3d), to butyric acid (Fig. 3e) and octanoic acid (a medium-chain fatty acid) induced stresses (Fig. 3f), and to ethanol stress (Fig. 3g). However, under the conditions used for susceptibility assays, increased tolerance to thermal-induced stress was not detected (Fig. 3h).

Considering the transcriptional factors exhibiting alterations at the transcriptional level in the evolved strain, only twenty six percent have a functional description (Table 4) that may be used to infer their potential functions. Based on this, the identified transcription factors appear to have roles in response to multiple stress conditions, including osmotic, oxidative, heat, weak acid and DNA replication stresses, which is in accordance with the transcription regulatory networks predicted through bioinformatics analyses. Interestingly, three of the transcription factors appear to encode heat shock transcription factors; two of them display increased transcript levels (IST_000711 and IST_001368) and one exhibits reduced transcript levels (IST_002508). IST_001368, presumed to have a copy number increase, has some similarities to the transcription factors *SFL1* (35% identity) and *SKN7* (31% identity) of *S. cerevisiae*, associated to the repression of flocculation related genes and activation of stress-responsive genes, and associated to the optimal induction of heat-shock genes in response to oxidative stress and osmoregulation, respectively. IST_002508 has some similarities to the *S. cerevisiae* transcription factor *MGA1* (27% identity), described to have a role in filamentous growth. IST_000711 does not display any significant similarities with other yeast species.

**Table 4:** Transcription factors displaying altered transcription levels in the evolved strain

ORF	LogFC	Missense variants	CNV	*R. toruloides* NP11 Orthologue	*S.cerevisiae* Orthologue	Description
IST_000711	1.30	1	–	RHTO_07168	–	Heat shock factor (HSF)-type transcription factor
IST_001231	− 1.06	0	–	RHTO_04639	–	–
IST_001368	2.09	0	Gain	–	–	Heat shock transcription factor
IST_001453	− 2.83	0	–	RHTO_02353	–	–
IST_001769	1.20	0	–	RHTO_02319	–	–
IST_002119	-2.07	1	–	RHTO_06229	*YOX1*	Homeobox transcriptional repressor; binds to Mcm1p and to early cell cycle boxes (ECBs) in the promoters of cell cycle-regulated genes expressed in M/G1 phase
IST_002508	− 1.28	0	–	RHTO_07521	–	Heat shock transcription factor
IST_002882	1.16	0	–	RHTO_00740	–	–
IST_004052	1.61	0	–	RHTO_01082	–	–
IST_004206	1.21	0	–	RHTO_07862	–	–
IST_004567	1.41	0	Gain	–	–	Nitrogen assimilation transcription factor (nit-4 *Neurospora crassa*; nirA *Aspergillus nidulans*)
IST_004648	1.66	0	–	RHTO_05651	*WAR1*	Binds to a weak acid response element to induce transcription of *PDR12* and *FUN34*
IST_004685	1.92	0	–	RHTO_05689	*PDR1*	Transcription factor that regulates the pleiotropic drug response
IST_005673	1.12	1	–	RHTO_05808	–	–
IST_006521	1.37	2	–	RHTO_06404	*COM2*	Transcription factor that binds *IME1* Upstream Activation Signal (UAS)ru
IST_007092	1.70	0	–	RHTO_07851	–	–
IST_007318	1.28	0	–	RHTO_00825	–	–
IST_007519	1.71	0	–	RHTO_07952	*SKO1*	Forms a complex with Tup1p and Cyc8p to both activate and repress transcription; cytosolic and nuclear protein involved in osmotic and oxidative stress responses
IST_007678	1.33	0	–	RHTO_05847	–	–

Genes predicted to encode transcription factors identified to have altered transcript levels in the evolved strain IST536 MM15 compared to the parental strain IST536. The orthologue genes in *S. cerevisiae* are displayed along with their functional description retrieved from the *Saccharomyces* Genome Database (SGD)—https://www.yeastgenome.org/. Descriptions, if available, are also provided for genes without *S. cerevisiae* orthologue. LogFC refers to the log_2_Fold-change values; CNV refers to copy number variations (“Gain” constitutes a copy number increase)

## Discussion

Comparative genomic and transcriptomic analyses of a multi-stress tolerant *R. toruloides* strain, evolved under selective pressure by increased methanol concentrations during growth with 5% (v/v) glycerol as the sole carbon source, versus the original strain, were conducted in yeast cells grown under non-stressful conditions. The objective was to infer possible functional basal alterations underlying the multi-stress tolerance phenotype in the evolved strain. Interestingly, the multi-stress tolerance phenotypes previously described for the evolved strain (towards methanol, acetic and formic acids, HMF, and furfural) [15] were extended in this study to hyperosmotic, saline, genotoxic and oxidative stresses, and stresses induced by ethanol, octanoic and butyric acids. These stress conditions were tested based on the indications obtained from the performed omics analyses. Remarkably, under the conditions used for the susceptibility test assays, either in this study or in the former study [15], the more marked phenotypes were registered for osmotic-, salt-, and ethanol-induced stresses. These stress conditions are of the same nature as the stress conditions employed during the ALE experiment (methanol and high glycerol concentration). Considering that the stresses imposed during the ALE experiment induce oxidative stress and mutagenesis, the other detected tolerance phenotypes in the evolved strain appeared likely. Several indications emerging from the performed genome-wide analyses support the notion of the existence of integrating mechanisms that sense and respond to different forms of sub-lethal stress as the foundation of the cross-tolerance phenomenon described herein [14].

The proposed convergence from a triploid genome in the parental *R. toruloides* strain to a diploid genome in the evolved strain presents a remarkable case of ploidy reduction. This phenomenon aligns with previous findings in various yeast species under diverse stress conditions [66], suggesting a broader evolutionary strategy among yeasts to adapt to environmental challenges through ploidy level adjustments. This genotypic shift may be attributed to several evolutionary pressures and genetic mechanisms. Different environmental conditions were noted to influence the fitness advantages associated with varying ploidy levels [17]. The presence of high methanol and glycerol concentrations may have created a unique selective landscape where the diploid state offered a survival or replication advantage over the triploid state. Triploids are often at a genomic disadvantage due to the challenges in maintaining stability during cell division [56]. Diploids, in contrast, have a more stable genome during cell division, which could contribute to higher cellular fitness [56]. Although the specific genetic mechanisms leading to the reduction in ploidy level are not clearly understood, previous studies have suggested that such reductions can occur through non-disjunction events during cell division or through selective chromosome loss mechanisms [56]. These processes could have been facilitated or accelerated under stressful conditions imposed on the yeast cells during the ALE experiment. Remarkably, the increased tolerance phenotypes observed for the evolved diploid strain appear to be stable over time.

The observed increase in transcriptional levels from genes encoding ferrireductases apparently suggests an increased requirement for iron in the evolved strain. Ferrireductases are essential enzymes for reducing Fe(III) to the more bioavailable form Fe(II) [48], indicating a potential adaptation to enhance iron acquisition based on their increased transcript levels. Fe–S clusters are vital for numerous cellular functions, including electron transport in mitochondria and the regulation of gene expression [30]. The observed increase in transcript levels from genes involved in the assembly of these Fe–S clusters may suggest yeast adaptation to higher energy demands under the stresses present during the ALE experiment. Moreover, proteins involved in DNA repair and replication often contain Fe–S clusters as essential cofactors [30]. The concurrent increase in transcript levels from Fe–S cluster assembly and DNA repair genes in the evolved strain suggests an integrative adaptation mechanism to the stresses imposed during ALE, supporting higher metabolic demands of cellular defence mechanisms and maintenance of genomic stability. Even in non-methylotrophic yeast cells, methanol is oxidized to formaldehyde intracellularly, a process catalysed by alcohol dehydrogenases (ADH) [38]. Formaldehyde is a highly cytotoxic compound (more than methanol) that disrupts DNA structure by reacting with DNA bases, causing mutations, strand breaks, and cross-linking, thereby hindering DNA replication and transcription, and affecting cell viability [24]. In the evolved strain, the observed basal enhancement of DNA repair mechanisms is likely a critical preventive adaptation mechanism to mitigate the toxic effects of methanol and formaldehyde, thereby preserving genomic integrity, a mechanism reported for *S. cerevisiae* [38]. In addition, the increased transcript levels and copy numbers from genes encoding ADHs and aldehyde dehydrogenases (ALD) in the evolved strain may also underlie the increased tolerance of the evolved strain to furfural and HMF stresses since these compounds inhibit both ADHs and ADLs, and negatively impact glycolytic and TCA fluxes [41].

The crucial role played by the cell wall in the adaptation and tolerance of yeasts to various biotechnologically relevant stresses has been thoroughly detailed in a recent review article [50]. The existence of integrating mechanisms that sense and respond to different forms of sub-lethal stress are the foundation of the cross-tolerance phenomenon [14]. The multi-stress tolerant evolved strain examined in this study, was obtained by ALE under conditions representing only a subset of the broader range of stress conditions to which this strain is tolerant. The integrated molecular responses here described provide the basis for the involvement of the cell wall in cross-stress protection. To sense, survive, and adapt to different stresses, yeasts rely on a network of signalling pathways that cooperate to tightly regulate the composition, organization and biophysical properties of the cell wall , which serves as the first line of defence against a wide range of environmental stresses [50]. The increased transcriptional levels from genes involved in cell wall biosynthesis and remodelling registered in the evolved strain suggest a coordinated alteration of the cell wall at the molecular and structural levels. The postulated enhanced synthesis of cell wall components like chitin, mannoproteins, and glucans suggested to occur in the evolved strain is expected to play a major role in maintaining cell wall integrity under stress [31, 50]. This is reinforced by the reported involvement of genes harbouring genomic variants in the evolved strain, in the biosynthesis of cell wall components with implication in stress tolerance. For instance, $\beta$-glucan biosynthesis and assembly encoding genes, *CWH41* and *EXG1*, are associated to methanol and thermotolerance, evidenced through a chemogenomic approach in *S. cerevisiae*, and, attributed to an SNP in the *Kluyveromyces marxianus* gene, respectively [50]. In addition, a chitin synthase encoding gene (*CHS1*) has been associated to acetic and ethanol tolerance, and *MNN2*, encoding a $\alpha$-mannosyltransferase was linked to acetic acid stress [50]. This cell wall remodeling response appears essential for limiting the entry of toxic compounds and enhancing overall cellular robustness against diverse stress effectors, including chemical inhibitors present in lignocellulosic hydrolysates, such as organic acids and furan aldehydes [32]. The dynamic remodelling of the cell wall also facilitates cellular adaptation to fluctuating external osmolarities [50]. Remarkably, the evolved strain was previously shown to possess a cell wall significantly less susceptible to zymolyase activity compared to the parental strain, suggesting a physiochemical different cell wall [15]. Furthermore, genomic variants in genes encoding key kinases that occupy a central position in cell wall integrity signalling in *S. cerevisiae* were identified, highlighting the substantial role of cell surface remodelling as a central adaptive response in the evolved strain. Although more specific responses may vary, the general trend of cell wall reinforcement and remodelling in the adaptive response to environmental stress appears to be conserved across different yeast species [50, 62] and this trend clearly emerges from this study.

The ability to change membrane lipid and protein composition is among the multitude of diverse mechanisms developed by yeasts to cope with environmental stresses in their natural environments and in industrial bioprocesses [10]. Plasma membrane efflux pumps, implicated in multidrug resistance (MDR), are considered essential for extruding toxic compounds from the cell interior and thus mitigating the deleterious effects of chemical stresses [52]. Engineering the expression and/or the amino acid sequences of these plasma membrane transporters has proved to have the potential for increasing yeast robustness towards bioprocess-related stress conditions [52]. Consistent with this concept, one substantial factor contributing to the acquisition of multi-stress tolerance in the evolved strain is the genomic and transcriptional alterations observed in genes encoding MDR transporters. This is the case of genes homologous to pleiotropic drug resistance ABC transporters, such as Pdr12, and Snq2 associated to the detoxification of lignocellulosic hydrolysates-derived inhibitors, such as weak acids and furan aldehydes [10, 34]. Moreover, MDR transporters of the major facilitator superfamily identified in this study (*AQR1*, *QDR1-3*, and *TPO1-3*) have been implicated in weak acid tolerance [52].

The inference of putative transcriptionally regulatory networks in the evolved strain compared with the original strain is instrumental in unveiling mechanisms underlying the acquisition of multi-stress tolerance. Relevant transcription factors were identified based on documented transcriptional regulatory associations in genes with orthologues in *S. cerevisiae*, focusing on the genes exhibiting increased transcriptional levels and without an increase in copy number. This bioinformatics inference of transcription factors indicated that the analysed genes were mainly implicated in response to oxidative, osmotic, and thermal stresses. Notable among the identified transcription factors identified were Met32, Pdr1/3, Rpn4, and Yap1. Yap1, known as a key regulator in oxidative stress response, has been implicated in the adaptation to furfural and HMF stresses [34], and methanol tolerance [38]. The synthesis of sulfur amino acids, essential to produce stress response proteins, is dependent on the transcription factor Met4, alongside its cofactors Met28/32 [54]. In addition, Rpn4 plays a crucial role as a transcriptional regulator within the ubiquitin-mediated proteasome degradation pathway, with its function extending to DNA repair and responses to ethanol and potentially methanol-induced stresses [38]. Under stress conditions, an interconnected regulatory circuit is formed through the cross-regulation between Rpn4, Pdr1/3, Yap1, and the heat shock transcription factor Hsf1, constituting a feed-forward regulatory gene network that may be part of a broader, highly coordinated gene interaction system responsive to stress [34]. This analysis is consistent with the transcription factors identified that exhibited increased transcriptional levels. Remarkably, several heat shock factor-type transcription regulators were registered, anticipated to play significant roles in the cellular responses to oxidative stress and osmoregulation, based on similarities with *S. cerevisiae*.

In addition, the transcription regulator Sko1 involved in the modulation of a transcriptional network upon osmotic stress also displayed increased transcriptional levels. Indeed, the evolved strain was shown herein to exhibit increased tolerance to oxidative stress and to sorbitol- and NaCl-induced osmotic stress compared to the parental strain, but not to thermal stress under similar test conditions. Other transcription factors included Pdr1 associated to the modulation of pleiotropic drug response, and Com2 and War1, both of which implicated in the cellular adaptation to short- and long-chain weak acids, respectively. It should be noted that gene expression regulation in basidiomycetes, in particular *R. toruloides*, is not well characterized [59]. Among the discernible differences between the transcriptional regulators of ascomycetes and basidiomycetes, it has been observed that regulators with more specialized functions tend to exhibit greater divergence suggesting potential differences in the complexity of gene regulation mechanisms between these fungal subgroups [59].

## Conclusions

This study provides insights into the genomic background and transcriptional profile of a multi-stress tolerant *R. toruloides* obtained by ALE, in comparison with its parental counterpart. These findings enlightened the molecular basis for multi-stress tolerance, advancing the knowledge of the mechanisms underlying the evolved strain heightened robustness. The advancement of functional characterization of *R. toruloides* genetic networks, along with the development and refinement of genetic tools, will be crucial in furthering our understanding of these genomic adaptations, paving the way for the rational improvement and application of this non-conventional yeast species in Biotechnology.

## Methods

### Yeast strains and growth conditions

The yeast strain *Rhodotorula toruloides* IST536 (PYCC 5615) and the derived mutant *R. toruloides* IST536 MM15, obtained through an Adaptive Laboratory Evolution (ALE) experiment [15], were used in this study. Strain IST536 is a conjugated strain derived from IFO 0559 x IFO 0880 [4]. IFO 0559 was originally isolated in Japan from wood-pulp, whereas IFO 0880 was originally isolated in Japan from the air [4]. Both IFO 0559 and IFO 0880 have genome sequences available under the accession numbers GCA_000988805.1 and GCA_000988875.2, respectively, and were previously regarded as haploids based on X-ray irradiation experiments [4]. The strains IST536 and IST536 MM15 were maintained at 4^∘^C in YPD agar plates (2% glucose (NZYtech), 1% yeast extract (Difco), 2% peptone (Difco), 2% agar). For long-term storage, the strains are preserved at -80^∘^C in YPD medium supplemented with 15% glycerol (v/v).

All pre-cultures were started from yeast isolated colonies grown on YPD agar plates and run in 100 mL shake flasks containing 50 mL of YPD medium, incubated at 30^∘^C, for 24 h with orbital shaking (250 rpm). For main yeast cultivation, pre-cultured cells were harvested by centrifugation at 4600 x g for 5 min at 4^∘^C, washed twice in sterile water and then inoculated in 20 mL of minimal medium (0.67% YNB (Difco), 2% glucose (NZYtech)) used in 100 mL shake flasks, at an initial OD_600_ of 1.0. Yeast growth was performed at 30^∘^C with orbital agitation (250 rpm) and monitored by measuring the optical density at $\lambda$= 600 nm (OD_600_) using a U-2000 spectrophotometer—HITACHI.

### Susceptibility assays

*R. toruloides* IST536 and IST536 MM15 cells were inoculated in 50 mL of YPD medium and grown overnight. Cells were then harvested and re-suspended in sterile water to a standardized optical density (OD_600_) value of 1.0. Serial tenfold dilutions (10^0^ to 10^4^) of these cell suspensions were spotted (4 $\mu$L) on YPD medium agar plates. For chemical susceptibility assays, YPD agar medium was supplemented with sorbitol (1.0 and 1.5 M), sodium chloride (NaCl; 0.8 and 1.0 M), methyl methanesulfonate (MMS; 0.005% and 0.010% (v/v)), hydrogen peroxide (H_2_O_2_; 1.5 and 2.0 mM), ethanol (6 and 7% (v/v)), butyric acid (C4; 5.0 and 8.0 mM), or octanoic acid (C8; 0.35 and 0.45 mM) and growth on agar plates was at 30^∘^C. For thermal tolerance assays YPD medium agar plates were incubated at 30^∘^C (control), 39^∘^C, and 40^∘^C. For weak acid stress assays (butyric and octanoic acid), the pH of the YPD agar medium was adjusted to 5.5. Growth on agar plates was recorded for up to 3 days. The results shown are representative of equivalent results obtained from at least two independent experiments.

### Genome sequencing, assembly and annotation

Genomic DNA from *R. toruloides* strains was isolated using the phenol–chloroform extraction method [21]. Genomic DNA was sequenced in an Illumina Novaseq 6000 platform, producing 2 x 150-bp paired-end reads. Library construction and sequencing were carried out by Novogene Bioinformatics Technology. The quality of the sequencing data was assessed using FastQC [1]. Paired-end reads were filtered for the presence of adapters, presence of undetermined bases (N > 10%), and quality (remove reads with Qscore $\le$ 5 that is over 20% of the total bases). Illumina sequencing resulted in 6,538,694 and 6,237,094 cleaned paired-end reads for IST536 and IST536 MM15, respectively.

Genomes were assembled using SPAdes v3.15 [3] with default settings. Coverage-versus-length (CVL) plots [12] were used to assess contaminants, resulting in the discard of scaffolds with coverage below 4x and length below 500 bp. The resulting assemblies were used as input for Redundans v2.0.1 [47] with default settings to generate an artificially reduced assembly by correcting for heterozygous regions and remove redundant contigs. The assembly completeness was estimated using Benchmarking Universal Single-Copy Orthologue (BUSCO)v5 tool with the *Basidiomycota* database [36], and assembly quality was analysed using Quality Assessment Tool for Genome Assemblies (QUAST) v5.2.0 [19]. Genome size, ploidy and heterozygosity were estimated using GenomeScope2.0 and Smudgeplot [49], and nQuire [61]. Final assembly statistics are reported in Additional File 1; Supplementary Table 1. The sequencing reads and assemblies for this study were deposited in the European Nucleotide Archive (ENA) at EMBL–EBI under accession number PRJEB71109 (https://www.ebi.ac.uk/ena/browser/view/PRJEB71109). Mitochondrial contigs were discerned through the analysis of coverage distribution across the IST536 assembly scaffolds using mosdepth v0.3.5 [44]. To eliminate nuclear regions and confirm mitochondrial regions, a NCBI BLAST+ blastn search was conducted. The resulting draft mitochondrial assembly underwent further refinement through read mapping of Illumina reads from IST536 using BWA–MEM v2.2.1 [28], followed by correction using Pilon v1.24 [60]. Subsequently, two distinct annotation methods were employed: MITOS2, with “RefSeq fungi” as the reference and “mold mitochondrial genetic code,” and AGORA, using the mitochondrial genome of *R. toruloides* NP11 (MT362617.1) as a reference. The circular map illustrating the draft mitochondrial genome was generated with OGDRAW.

Gene prediction and annotation for the complete genome assembly was performed with Funannotate v1.8.15 [43] pipeline, which includes masking of repetitive elements (RepeatModeler v2.0.5 and RepeatMasker v4.1.5 tools), *ab initio* gene-prediction training, using Augustus, and incorporation of transcript information based on mapped RNAseq data (accession number GSE254308) using HISAT2 v2.2.0 [26], with subsequent identification of transcripts using Stringtie v1.3.3 [45]. Functional annotation was inferred using Orthofinder v2.5.5 [13] to retrieve orthologues in *R. toruloides* NP11 (*Rhodotorula* spp. reference genome), IFO 0880 and IFO 0559 (IST536 ancestral strains), and *Saccharomyces cerevisiae* S288c.

### Variant calling and copy number variants

Read mapping was conducted using BWA–MEM v2.2.1 [28] with default parameters. Picard Toolkit https://broadinstitute.github.io/picard/ was used to mark duplicates with MarkDuplicates. Variant calling was performed with Mutect2 from the Genome Analysis Tool Kit (GATK) [2], where the original strain (IST536) was used as reference and set as the “normal” sample, whereas the evolved strain (IST536 MM15) was set as the “tumor” sample. Variants were annotated using SnpEff, filtering the missense and nonsense variants with SnpSift [9]. The determination of genes affected by copy number variations (CNVs), was conducted using a read depth coverage approach. The reads from IST536 and IST536 MM15 were aligned to the IST536 genome using BWA–MEM v2.2.1 [28]. The relative depth, hereafter referred to as the ratio of the read depth for each gene relative to the median read depth per gene across all nuclear scaffolds for each strain (excluding the mitochondrial scaffold and abnormally high coverage scaffolds), was calculated using mosdepth v0.3.5 [44]. Gain or loss of copy number was defined by calculating the log_2_ ratio between the relative depths of IST536 MM15 and IST536. A copy number increase was defined as log_2_ ratios above 1, whereas a copy number decrease was defined as ratios below -1.

### Sample RNA extraction, RNA-Seq, and differential expression analysis

RNA extraction was performed using a modified hot phenol method [35]. Purified DNA-free RNA samples were obtained using the commercial kit *RNA Clean & Concentrator*^TM^*-5* (Zymo Research). RNA sample quantitation, integrity and purity were assessed on the Agilent Bioanalyzer 5400 system. Messenger RNA was purified from total RNA using poly-T oligo-attached magnetic beads. After fragmentation, the first strand cDNA was synthesized using random hexamer primers followed by the second strand cDNA synthesis. The library was ready after end repair, A-tailing, adapter ligation, size selection, amplification, and purification. The library was checked with Qubit and real-time PCR for quantification and bioanalyzer for size distribution detection. Library preparations were sequenced on an Illumina platform and 125 bp/150 bp paired-end reads were generated.

Reads containing adapter sequences, containing poly-N, or low quality reads were removed from raw data (raw reads in fastq format) using Trimmomatic [5]. The quality-scores and GC content were then calculated using FastQC [1]. The genome of *Rhodotorula toruloides* IST536 was used as reference. Index of the reference genome (IST536) was built and paired-end clean reads were aligned using HISAT2 v2.2.0 [26]. Read counts that mapped to each gene were calculated using featureCounts from the Subread package v2.0.4 [29]. Differential expression analysis was performed using the DESeq2 R package v1.42.0 [33]. The p-values were adjusted using the Benjamini and Hochberg method. Corrected p-values of 0.05 and absolute fold-change of 2 were set as the threshold for significantly differential expression. The analysis of the differentially expressed genes (DEGs) was performed using the EuKaryotic Orthologous Groups (KOG) gene functional annotations for *R. toruloides* NP11, IFO 0550 and IFO 0880 strain based on the inferred orthology associations. The transcriptomics data discussed in this publication were deposited in NCBI’s Gene Expression Omnibus and are accessible through GEO Series accession number GSE254308 (https://www.ncbi.nlm.nih.gov/geo/query/acc.cgi?acc=GSE254308).

### Putative transcription regulators identification

Differentially expressed genes (DEGs) exhibiting increased mRNA levels and absence of copy number alterations in the evolved strain versus the original strain were selected for eventual identification of transcription factors. Two approaches were employed: one for searching what DNA-binding motif sequences were enriched in the selected genes promoter sequences, and other making use of the YEASTRACT+ portal [58] documented transcriptional regulatory associations to search for putative transcriptional regulators of the selected genes that have orthologues in *S. cerevisiae*.

For the first analysis, the promoter sequences of the selected genes were extracted as the − 1000 base pairs upstream the coding sequences. To analyse these promoter sequences, the HOMER (Hypergeometric Optimization of Motif EnRichment) software suite [20] was employed, using the complete set of *R. toruloides* IST536 promoter sequences as a background. A pairwise comparison of all identified motifs was carried out based on the Pearson correlation coefficient (PCC) and their average means as scoring strategy. This comparative analysis was executed utilizing the “compare_motifs” function from the universalmotif R package. Motifs displaying a PCC greater than 0.8 were then subjected to alignment with motifs from the JASPAR2022_CORE_non-redundant-fungi database. This alignment was performed using the TOMTOM tool within the MEME suite [18], enabling the identification of putative transcriptional factors associated with the observed regulatory motifs. The orthologous genes in *R. toruloides* IST536 corresponding to the identified putative *S. cerevisiae* transcriptional factors were obtained using Orthofinder v2.5.5 [13]. The tool *RankbyTF* from Yeastract was used to systematically rank the identified transcription factors.

For the second analysis, the selected *R. toruloides* genes with orthologues in *S. cerevisiae* were used directly as input for the tool *RankbyTF* from Yeastract database [58], against all *S. cerevisiae* transcription factors, and considering the transcriptional regulatory associations documented in the database for both unstressed and stressed conditions. The identification of putative transcription factors was made based on their ranking, modelled by a hypergeometric distribution and Bonferroni correction of the obtained p-value.

### Supplementary information

**Additional file 1: Supplementary Table 1.** Genome assembly statistics for* R. toruloides* IST536 and IST536 MM15 strains.** Supplementary Fig. 1.** Circular map of the* R. toruloides* IST536 draft mitochondrial genome generated by OGDRAW software.** Supplementary Fig. 2.** Categorical distribution and genomic regions affected by the variants identified.** Supplementary Fig. 3.** Ploidy analysis using nQuire.

**Additional file 2:** IST536 MM15 gene missense and non-sense variants.

**Additional file 3:** IST536 MM15 gene copy number variants (CNVs).

**Additional file 4:** Differential expressed genes (DEG) in IST536 MM15 compared with IST536 under non-stressing conditions.

## Data Availability

All data generated or analysed during this study are included in this published article and its supplementary information files (Additional files 1, 2, 3, and 4). The sequencing reads and assemblies for this study were deposited in the European Nucleotide Archive (ENA) at EMBL-EBI under accession number PRJEB71109 (https://www.ebi.ac.uk/ena/browser/view/PRJEB71109). The transcriptomics data discussed in this publication were deposited in NCBI’s Gene Expression Omnibus and are accessible through GEO Series accession number GSE254308 (https://www.ncbi.nlm.nih.gov/geo/query/acc.cgi?acc=GSE254308).
