# Supplementary material for: Cell envelope and stress-responsive pathways underlie an evolved oleaginous Rhodotorula toruloides strain multi-stress tolerance
Source: Biotechnol Biofuels Bioprod. 2024 May 28;17:71. doi: 10.1186/s13068-024-02518-0 (PMC11134681; doi:10.1186/s13068-024-02518-0)
Supplement: Supplementary file 1 — Additional file 1: Supplementary Table 1. Genome assembly statistics for R. toruloides IST536 and IST536 MM15 strains. Supplementary Fig. 1. Circular map of the R. toruloides IST536 draft mitochondrial genome generated by OGDRAW software. Supplementary Fig. 2. Categorical distribution and genomic regions affected by the variants identified. Supplementary Fig. 3. Ploidy analysis using nQuire. [file 13068_2024_2518_MOESM1_ESM.pdf]

Additional File 1

Table 1: Genome assembly statistics for *R. toruloides* IST536 and IST536 MM15 strains.

**Organism**	*Rhodotorula toruloides*	*Rhodotorula toruloides*
Strain	IST536	IST536 MM15
**Assembly**		
Assembly size (Mbp)	23.47	22.84
Number of contigs	374	317
N50 contig length (kbp)	138	169
Number of scaffolds	275	229
N50 scaffold length (kbp)	191	240
Average coverage depth	75X	72X
GC content (%)	62	62
**BUSCO Completeness (Basidiomycota)**		
Complete BUSCOs	88.1%	87.9%
Complete and single-copy BUSCOs	79.4%	81.2%
Complete and duplicated BUSCOs	8.7%	6.7%
Fragmented BUSCOs	2.8%	2.9%
Missing BUSCOs	9.1%	9.2%
Total BUSCO groups searched	1764	1764

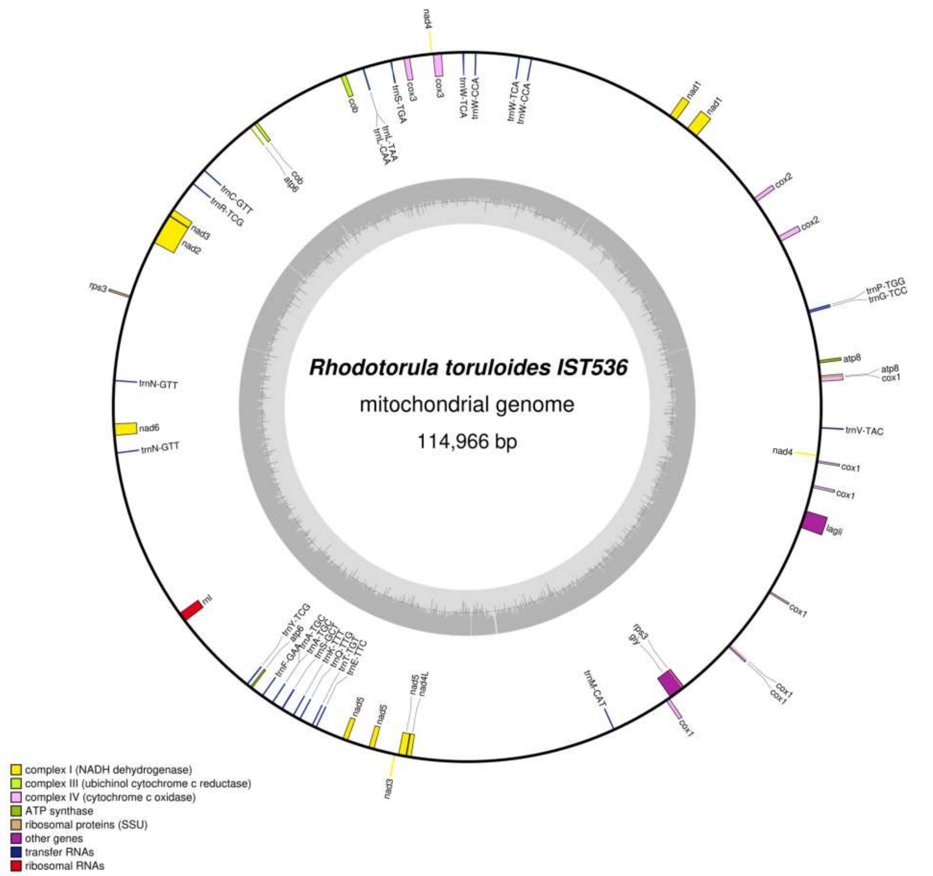

Fig. 1: **Circular map of the *R. toruloides* IST536 draft mitochondrial genome generated by OGDRAW software**. Genes and features are represented with different colour blocks. Genes with the same designation refer to exons. GC content graph is displayed in grey.

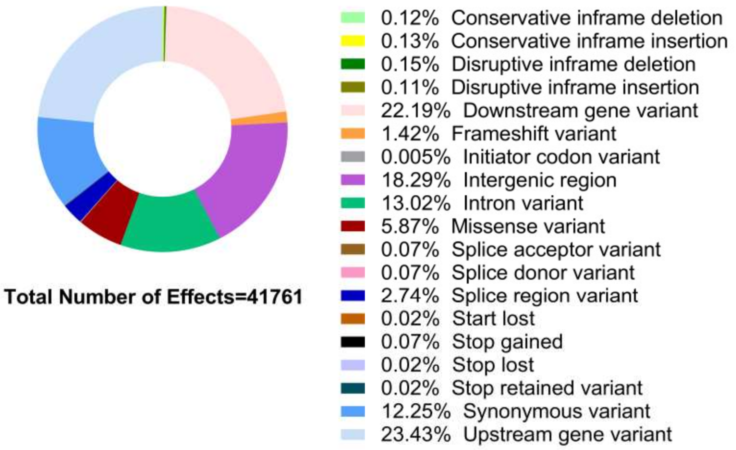

Fig. 2: **Categorical distribution and genomic regions affected by the variants identified**. Effects and genomic regions of variants in the evolved strain IST536 MM15 compared to the parental strain IST536 were annotated using snpEff.

A

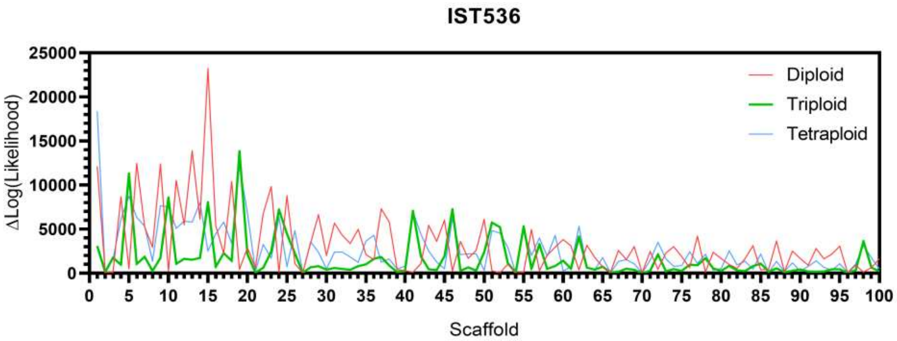

B

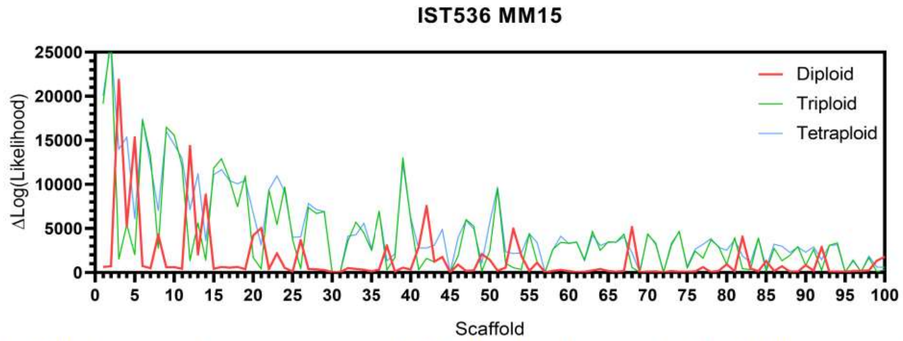

Fig. 3: **Ploidy analysis using nQuire**. Plots display the ΔLog(Likelihood) for the first 100 scaffolds of each genome assembly, *R. toruloides* IST536 (**A**) and IST536 MM15 (**B**).
